# Deep Learning Based Superconducting Radio-Frequency Cavity Fault Classification at Jefferson Laboratory

**DOI:** 10.3389/frai.2021.718950

**Published:** 2022-01-03

**Authors:** Lasitha Vidyaratne, Adam Carpenter, Tom Powers, Chris Tennant, Khan M. Iftekharuddin, Md Monibor Rahman, Anna S. Shabalina

**Affiliations:** ^1^ Jefferson Laboratory, Newport News, VA, United States; ^2^ ODU Vision Lab, Department of Electrical and Computer Engineering, Old Dominion University, Norfolk, VA, United States; ^3^ Jefferson Laboratory, Warrington, United Kingdom

**Keywords:** time-series classification, fault identification, superconducting radio-frequency cavities, particle accelerator, LINAC, deep recurrent learning, convolutional neural networks

## Abstract

This work investigates the efficacy of deep learning (DL) for classifying C100 superconducting radio-frequency (SRF) cavity faults in the Continuous Electron Beam Accelerator Facility (CEBAF) at Jefferson Lab. CEBAF is a large, high-power continuous wave recirculating linac that utilizes 418 SRF cavities to accelerate electrons up to 12 GeV. Recent upgrades to CEBAF include installation of 11 new cryomodules (88 cavities) equipped with a low-level RF system that records RF time-series data from each cavity at the onset of an RF failure. Typically, subject matter experts (SME) analyze this data to determine the fault type and identify the cavity of origin. This information is subsequently utilized to identify failure trends and to implement corrective measures on the offending cavity. Manual inspection of large-scale, time-series data, generated by frequent system failures is tedious and time consuming, and thereby motivates the use of machine learning (ML) to automate the task. This study extends work on a previously developed system based on traditional ML methods (Tennant and Carpenter and Powers and Shabalina Solopova and Vidyaratne and Iftekharuddin, Phys. Rev. Accel. Beams, 2020, 23, 114601), and investigates the effectiveness of deep learning approaches. The transition to a DL model is driven by the goal of developing a system with sufficiently fast inference that it could be used to predict a fault event and take actionable information before the onset (on the order of a few hundred milliseconds). Because features are learned, rather than explicitly computed, DL offers a potential advantage over traditional ML. Specifically, two seminal DL architecture types are explored: deep recurrent neural networks (RNN) and deep convolutional neural networks (CNN). We provide a detailed analysis on the performance of individual models using an RF waveform dataset built from past operational runs of CEBAF. In particular, the performance of RNN models incorporating long short-term memory (LSTM) are analyzed along with the CNN performance. Furthermore, comparing these DL models with a state-of-the-art fault ML model shows that DL architectures obtain similar performance for cavity identification, do not perform quite as well for fault classification, but provide an advantage in inference speed.

## Introduction and Motivation

**FIGURE 1:**
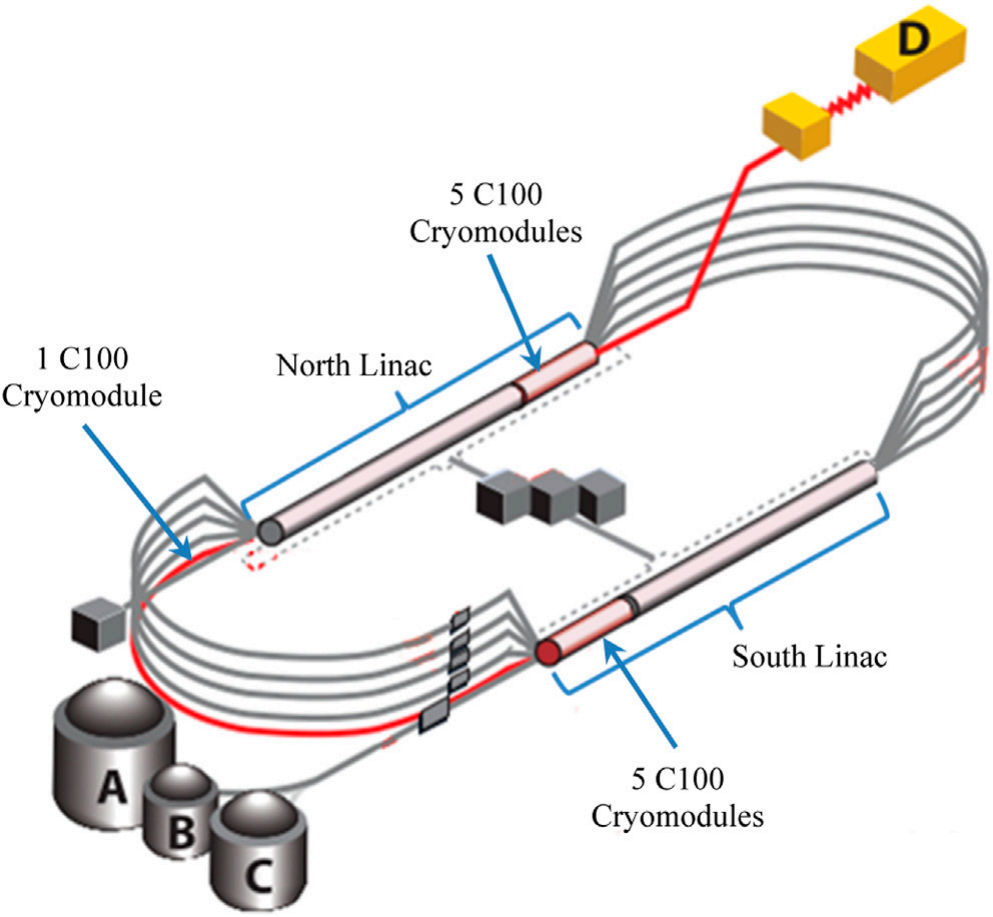
A schematic of CEBAF with the experimental halls (A, B, C, and D), indicating the locations of the C100 cryomodules used for this study.

The Continuous Electron Beam Accelerator Facility (CEBAF) is a high power, continuous wave recirculating linac capable of servicing four different experimental nuclear physics end stations simultaneously (Reece, 2016). Recently, CEBAF underwent an energy upgrade program to reach a peak energy of 12 GeV. The upgrade included the installation of 11 additional cryomodules—each capable of 100 MV energy gain and denoted as C100s. Each cryomodule is comprised of 8 superconducting radio-frequency (SRF) cavities. Figure 1 shows a schematic of CEBAF along with locations of the C100 cryomodules. Additionally, a digital low-level radio frequency system (LLRF) is developed to control the new cryomodules.

At high energy, when cavity gradients are being pushed to their limits, CEBAF experiences frequent short machine downtime trips (events that can be resolved within 5 min) caused by numerous SRF system faults. Specifically, CEBAF operational runs in 2019 observed an average of 4.1 RF downtime trips per hour, culminating in approximately 1 h of beam time lost each day. The primary means of reducing the RF trip rate is to lower the accelerating gradient of problematic cavities, which in turn hinders CEBAF’s ability to reach 12 GeV.

Approximately 25% of these RF faults originated from the newly deployed C100 cryomodules. However, each C100 fault takes significantly longer to recover than their older counterparts. Determining which C100 cavity is associated with a fault and the underlying cause of the fault is not trivial. Typically, this work requires subject matter expertise, is time consuming, and is performed days or weeks after the events. In the interim, accelerator operators do their best to reduce trip rates through gradient reduction. However, incorrectly identifying the fault type makes it impossible to address the root cause, while incorrect cavity identification can lead to unnecessary reductions in cavity accelerating gradients.

**FIGURE 2:**
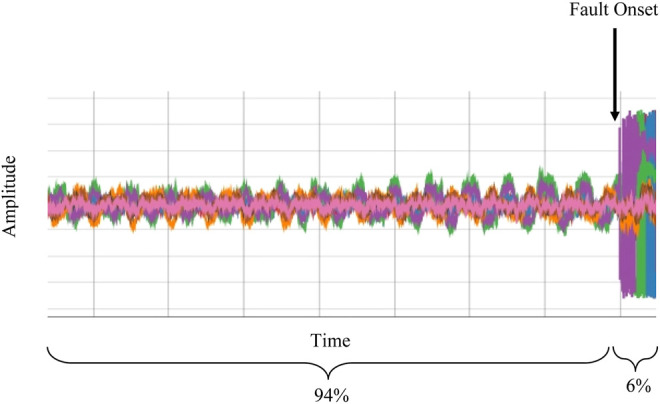
An example waveform segment captured by the DAQ system. The total duration of the captured waveform is 1.64 s.

In order to study the nature of these faults, we have implemented a new data acquisition system (DAQ), leveraging the digital LLRF system of the C100 cryomodules. The DAQ system is configured to capture 17 RF waveforms originating from each cavity in the 11 C100 cryomodules. The system is triggered at the onset of an RF failure, and a 1.64 s synchronized segment of the waveforms is retained for analysis (8,192 samples at 5 kHz). The system is configured such that 94% represents pre-fault data, while the remaining 6% is post-fault. This is illustrated in Figure 2.

Parameters such as sampling frequency and the ratio of pre-fault to post-fault data in the captured waveforms are established by subject matter experts (SMEs) such that the data contain sufficient information to determine the nature of an RF failure event (Tennant et al., 2020). Supplementary Appendix Table SA1 in Appendix A describes the 17 recorded RF signals. Typically, a subset of waveforms from each cavity in a cryomodule is inspected manually by SMEs to determine: 1) the identity of the cavity which faulted first, and 2) the type of fault. A typical operational run of CEBAF spans several weeks, and with the current RF downtime trip rate of 4.1/hr, our DAQ system collects data from several thousand events. The sheer number of waveforms and the subtlety of how certain faults manifest themselves make this manual inspection process laborious. Moreover, inspecting the data is usually conducted post-run due to the time-consuming nature of the process. Therefore, corrective measures can only be applied in the subsequent operational run. A fast RF cavity fault classification system (e.g. results reported seconds after a fault) enables CEBAF operators to apply corrective measures within the same operational run, thereby reducing unnecessary downtime.

A state-of-the-art ML based fault classifier model is currently deployed for use in CEBAF (Tennant et al., 2020). Many classical machine learning (ML) methods have been developed and utilized for time-series analysis in a variety of domains such as economics (Beveridge and Nelson, 1981; Caiado et al., 2006), the social sciences (Meidinger, 1980; Shumway et al., 2000), and healthcare (Chua et al., 2010; Shoeb and Guttag, 2010). While the present ML solution is adequate for our current “human-in-the-loop” paradigm, there is a desire to move toward an autonomous, fault prediction system where preventative action is taken before the fault occurs. Initial work suggests that the window of opportunity to predict an impending fault and effect a change to the system is a few hundred milliseconds (Vidyaratne et al., 2021). Therefore, to meet the stringent time constraints model inference times should be as fast as possible. Typical ML modeling for time-series analysis requires developing a pipeline of appropriate feature engineering, selection, and classification schemes (see Supplementary Appendix SAB). Consequently, such classical ML pipelines are inherently limited by the use of human expertise and intuition at each step of the learning process and by the need to explicitly compute features—which represents a potential bottleneck. To mitigate such limitations, this work investigates the performance of deep learning (DL) based methods.

*Data* of this paper provides details of the dataset and the preprocessing steps applied. *Deep Learning Models* discusses the deep recurrent learning (DRL) and convolutional neural network (CNN) architectures used in our study, while *Results and Discussion* describes the training methods and results for each, as well as a comparison to the performance of the ML model. *Summary and Conclusion* provides a summary of the work.

## Data

The dataset used in this study is curated using samples retrieved from CEBAF operational runs between the spring of 2019 and the fall of 2020. Each data sample consists of 17 time-series waveforms from each cavity of a given cryomodule. The data samples are inspected and annotated by an SME with labels corresponding to the identity of the cavity that faulted first and the type of fault. Note that the data collection for this project spanned several years over many CEBAF operational runs. The SME has routinely inspected the data, and over time has converged on 8 distinct RF fault types that can occur in the CEBAF system. The data used for this study is specifically selected from the latest operational runs.

**FIGURE 3:**
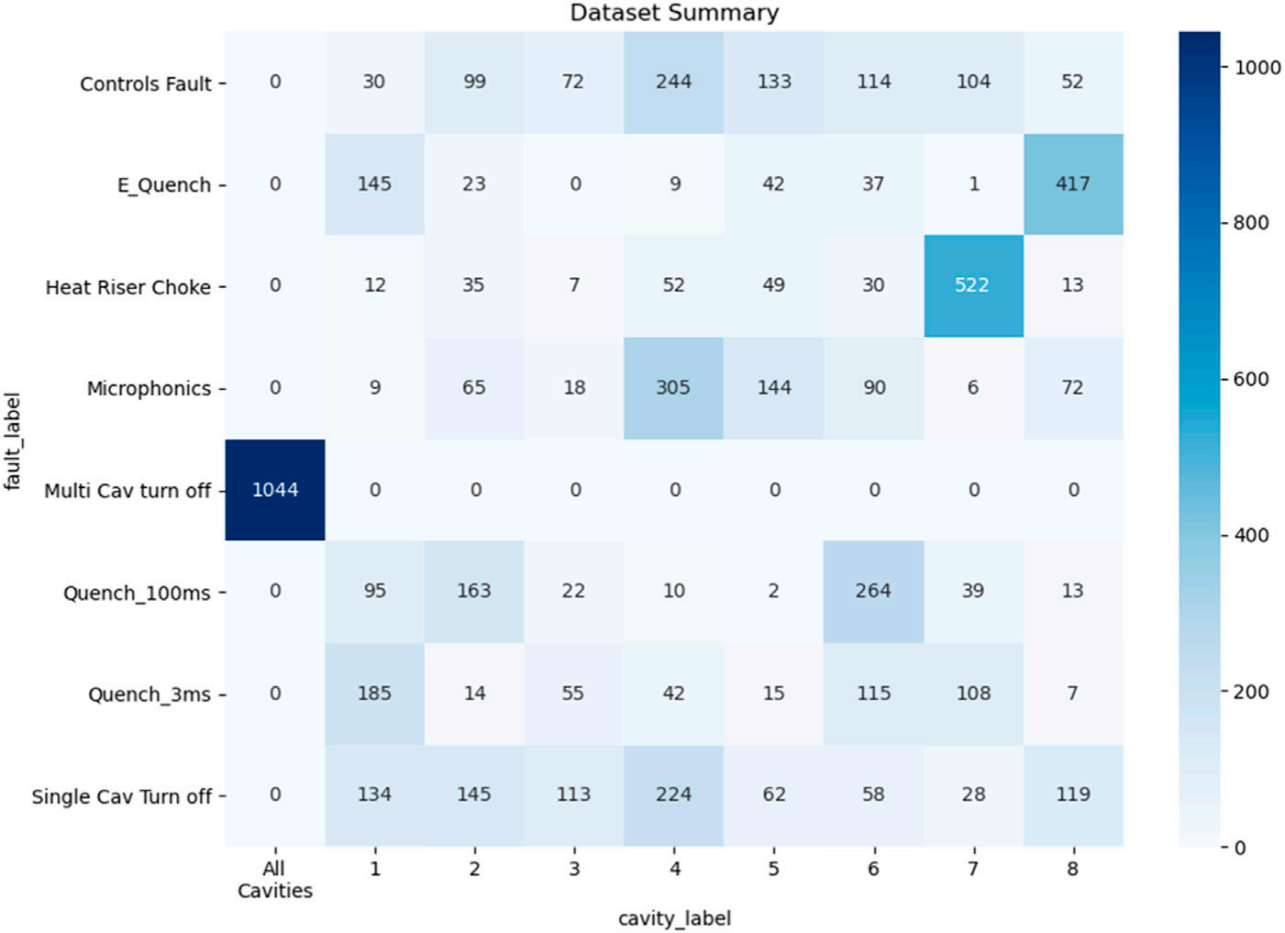
Dataset representation cavity identification, and fault classification tasks.

The dataset utilized for training, validation and testing of the deep learning models consists of 6,027 events. The class-wise itemization of the dataset are shown in Figure 3. In addition to using all 17 waveforms per cavity, we also experiment using just 4 waveforms (GMES, GASK, CRFP, DETA2), identified by SMEs as having the greatest predictive power.

Note from Figure 3 that the fault class named “Multi cavity Turn Off” is directly correlated with the “All cavities” class in the cavity identification task. Nevertheless, we treat cavity identification and fault classification as separate tasks at this stage of the study in an effort to build independent and generalized models.

### Preprocessing

Time-series data is a sequence of values obtained through observations over time. The sequence of observations are usually evenly spaced temporally and commonly represented (Malhotra et al., 2015) as a vector, $X=\left\{x^{\left(1\right)},x^{\left(2\right)},\ldots ,x^{\left(n\right)}\right\}$ where each element $x^{\left(t\right)}\in R^{m}$ of X is a vector of m values such that $x^{\left(t\right)}=\;\left\{x_{1}^{\left(t\right)},\;x_{2}^{\left(t\right)},\;\ldots ,\;x_{m}^{\left(t\right)}\right\}$ . The size of m is determined by the dimensionality of input at time t , and may have an impact on the processing speed based on the algorithm used. The dimensionality of each individual cavity RF waveform is singular, i.e. m=1 .

The values within the raw signal waveforms feature large variations (orders of magnitude) between cavities and among signal types within individual cavities. Even the same waveform from the same cavity may be at different absolute values from fault to fault, as parameters like the gradient set point change between faults. However, fault behavior as exhibited by waveform is qualitatively similar relative to each other within a fault example. Accordingly, we apply time-series standardization using the *z*-score technique on each individual waveform from an individual fault example based on its own mean and standard deviation. No population-level standardization is applied. The *z*-score function applied to each time-series waveform X is given in Eq. (1)

$$X_{norm}=\frac{X-\mu }{\sigma }$$ (1)

where μ and σ are the mean and the standard deviation values of X, respectively. This standardization is applied to each waveform individually as an initial preprocessing step common to all models. Additionally, we apply periodical down sampling to each signal in order to obtain a shorter signal length with a manageable number of time steps. We experiment with down sampling factors of 16 and 32 for deep recurrent learning models (see *Deep Recurrent Learning*) and factors of 8, 16, and 32 for convolutional neural network models (see *Convolutional Neural Network*).

## Deep Learning Models

Deep learning models are essentially large-scale artificial neural networks that have the ability to learn complex tasks through examples. Hence, DL models effectively mitigate the limitations of ML pipelines by simultaneously learning the feature extraction, feature selection and classification steps through neural layers without human intervention.

This work investigates the efficacy of models based on two prominent DL architectures: deep recurrent learning and a convolutional neural network. Deep recurrent learning is based on long short-term memory (LSTM) layers which have the ability to learn both long-term and short-term temporal features. Additionally, the bi-directional functionality allows the LSTM to process time-series in the forward and backward directions simultaneously. This further enhances the ability of LSTM to retain patterns encountered at different locations of the time-series signal using both past and future contexts (Schuster and Paliwal, 1997). Convolutional neural networks, on the other hand, are adept at learning spatial dependencies in an image through the use of multiple trainable filters while maintaining efficiency with weight sharing. While CNNs have achieved state-of-the-art performance in many image processing and computer vision applications (LeCun et al., 1998; Krizhevsky et al., 2012; Simonyan and Zisserman, 2014), the data in this study consists of multiple RF signals that have high temporal, but little spatial, relevance. Therefore, it is necessary to apply a transformation to represent time-series data in a 2 dimensional image suitable for input to a CNN. A straightforward approach is to reconstruct waveforms directly in a one dimensional (Ullah et al., 2018; Eren et al., 2019) or multi-dimensional (Wei et al., 2018; Zhao et al., 2019; Cho and Hwang, 2020) array, which we refer to as direct signal conversion (DSC) and which is discussed more fully in *Convolutional Neural Network*.

### Deep Recurrent Learning

**FIGURE 4:**
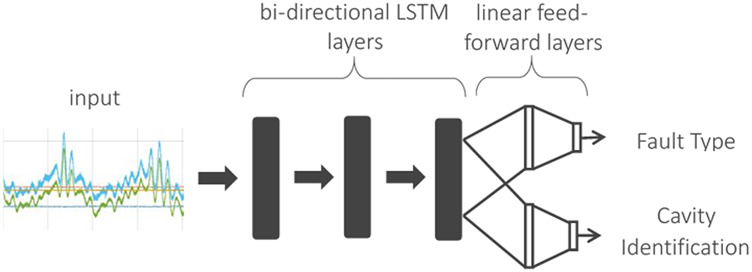
Deep LSTM branched classification model.

A schematic of the DRL model architecture is shown in Figure 4. The first LSTM layer is configured to accept either 136 (17 signals per cavity × 8 cavities) or 32 (4 signals per cavity × 8 cavities) time-series signals. The model contains three bi-directional LSTM layers, each with 64 feature dimensionality at the front end for feature learning. The back end of the model is a branched architecture with multiple feed-forward neural layers to enable simultaneous learning of both cavity and fault identification tasks. That is, the features learned by LSTMs are shared among the two paths to perform both tasks in a computationally efficient end-to-end trainable manner. The deep LSTM classification model is fully implemented in Python utilizing the PyTorch deep learning library (Paszke, 2019).

End-to-end training for both tasks is achieved using two different cost functions simultaneously. Note that cavity and fault recognition tasks for this model are posed as a 9-class and 8-class classification problem, respectively (see Figure 3). Consequently, we use categorical cross-entropy (Géron, 2019) as our cost function for both tasks. The final cost to be optimized is a linear combination of individual costs as follows:

$$Final_{cost}={\text{cavity}}_{cost}+{\text{fault}}_{cost}$$ (2)

Note that we do not explicitly weight the individual costs as preliminary experiments found no gain in overall performance by doing so. We utilize the Adam optimization algorithm (Kingma and Ba, 2014) as the weight update scheme for the entire network, with a learning rate of 0.01 and a weight decay rate (for weight regularization) of 0.99. The network is trained for 50 epochs.

### Convolutional Neural Network

**FIGURE 5:**
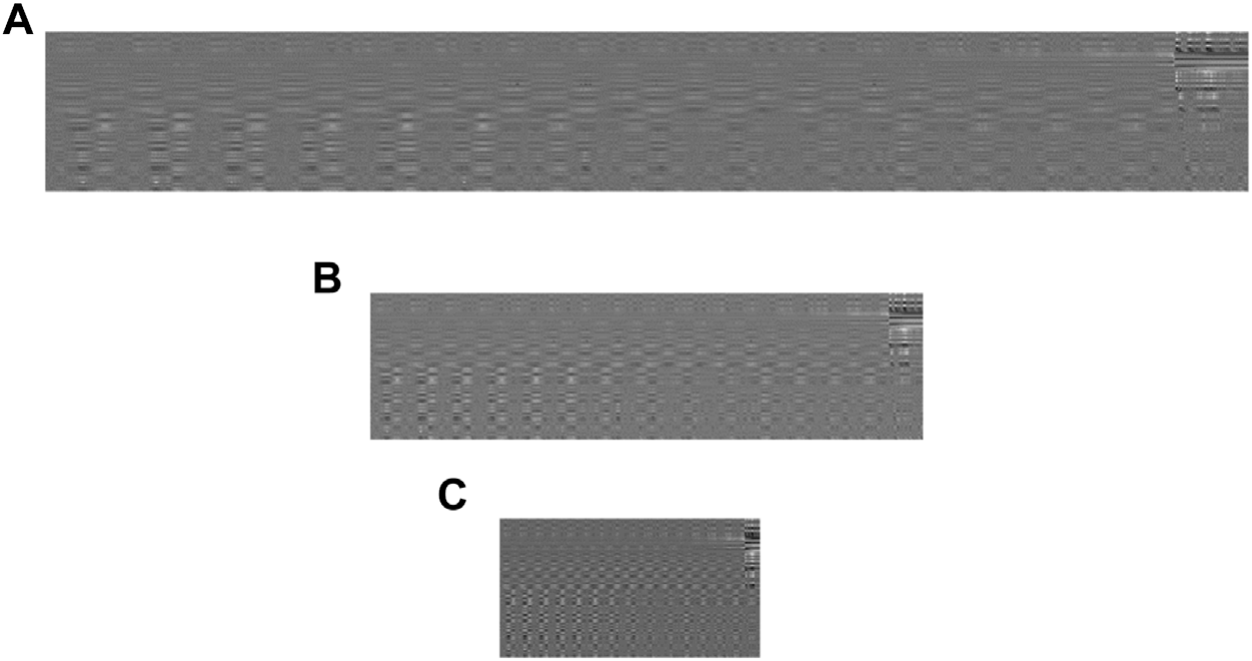
Example raw time-series direct conversion as 2D images for a Quench 100 ms fault type. **(A)** image representation of size 136 × 1,024, **(B)** image representation of size 136 × 512, **(C)** image representation of size 136 × 256.

The time-series data we use for this study needs to be suitably represented in a multidimensional tensor format compatible for a typical CNN input. A straightforward representation of the signals as a 2-dimensional image is achieved simply by considering time as the second dimension. The total number of RF signals for each data sample (17 signals/cavity × 8 cavities = 136 ) are arranged as rows in the representation, and the columns correspond to time in the signals after standardization and down sampling to obtain a more amenable rectangular 2D array. Note that the SRF cavities in cryomodules are arranged sequentially. The direct conversion representation is designed to preserve this relative physical locality of cavities by arranging signals from cavities in the same sequence. Additionally, we maintain the same arrangement of signal types (signals 1 through 17) for each cavity and apply filters of stride 1 for all convolutional layers assuming that significant correlated activity in waveforms extend across neighboring cavities. Consequently, this arrangement preserves potential correlations across signals within a cavity, as well as some signals of neighboring cavities. We experiment with down sampling factors of 8, 16, and 32 to obtain 2D arrays of size 136 × 1,024, 136 × 512, and 136 × 256. An example representation obtained by this method is displayed in Figure 5. While down sampling factors 16 and 32 yield input sizes that directly match that of the DRL model, the computational efficiency of CNN permits an even larger input size with a lower down sampling factor of 8. We utilize this capability to analyze the performance for input with more time samples.

**FIGURE 6:**
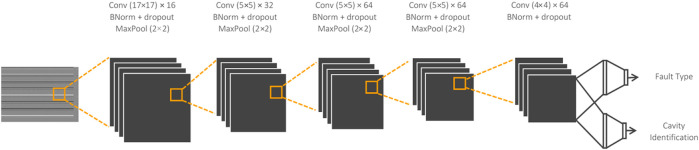
CNN architectures for RF signal direct converted input.

The CNN is developed to process the input arrays and simultaneously classify the cavity and the fault type. This is achieved using a fully connected, branched architecture at the back end. The front end of the model consists of 5 convolutional layers, each followed by batch normalization, and dropout (rate = 0.5). Each layer is activated using the leaky ReLU function. The detailed architecture shown in Figure 6.

Note that the architecture in Figure 6 contains 2D filters of size 17 × 17 in the first layer. This filter size is deliberately set to ensure that the layer’s receptive field overlaps all 17 signals coming from each cavity at certain convolutional intervals, corresponding to the signal arrangement process of the direct conversion method. Consequently, the 2D filters enable the CNN to capture potential correlation patterns across signals within a cavity, and also across neighboring cavities.

Similar to the DRL, the CNN model architecture enables end-to-end training for both tasks using two different cost functions simultaneously. We utilize the same cost as defined in Eq. 2. Additionally, we utilize the Adam optimization algorithm as the weight update scheme for the entire network, with a learning rate of 0.001 and a weight decay rate (for weight regularization) of 0.99. The network is trained for 400 epochs.

## Results and Discussion

We report quantitative performance figures for each DL system, and a performance comparison with the currently deployed ML pipeline (Tennant et al., 2020). Following the typical DL workflow, the training and testing of the DL models are carried out using a data split of 60% (3,616 events) for training, 20% (1,205 events) for validation, and 20% (1,206 events) for testing (stratified). Note that the same 1,206 testing events are used to evaluate all models to conduct a fair testing data performance comparison.

### Deep Recurrent Learning

**FIGURE 7:**
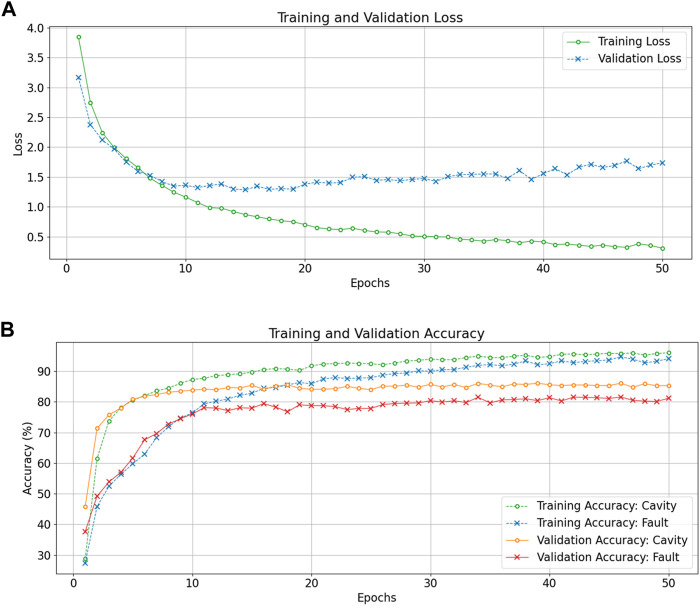
Training and validation loss curves **(A)**, and training and validation accuracy curves **(B)** as a function of the epochs for the DRL classifier processing input data of 32 signal × 256 time steps.

The training is performed for 50 epochs with batch-wise gradient updates. We obtain validation results at each epoch to verify proper training. Testing is only performed once the network is fully trained. We report training, validation, and testing accuracies for each experiment. Figure 7 shows loss and accuracy (number of correct classifications/total number of classifications) curves for DRL training and validation with input size: 32 signals × 256 time steps.

The training and validation loss curves in Figure 7A (top plot) show that the DRL converges to a minimum quickly, albeit with signs of slight overfitting. However, the accuracy curves in Figure 7B (bottom plot) show the validation accuracies are quite stable for both tasks throughout the 50 epoch training duration. We also observe that the DRL quickly obtains high accuracy for the cavity classification task (roughly 80% after 5 epochs) while fault classification requires more training epochs to obtain a stable accuracy (80% is reached after 30 epochs). This indicates fault classification is a significantly more complex task than cavity identification.

We report and analyze the performance of the DRL model with three different input data combinations. In the first, denoted as DRL_1_, we utilize an input comprised of all 136 signals (17 signals from each cavity × 8 cavities) per sample with signals down sampled by a factor of 32 (input size: 136 × 256). In the second (DRL_2_), we use an input combination of 32 signals (4 signals from each cavity × 8 cavities) per sample with signals down sampled by the same factor of 32 (input size: 32 × 256). Thirdly (DRL_3_), we use an input with the same 32 signals but using a lower down sampling factor of 16 (input size: 32 × 512). Table 1 summarizes the classification results of both cavity and fault recognition tasks obtained using 3,616 training, 1,205 validation, and 1,206 testing events. Note that the testing dataset is commonly used to evaluate all models in this study.

**TABLE 1:** Results of deep recurrent classification model performance after 50 epochs.

Model	Input combination	DRL input size	Cavity identification		Fault classification	
			Train/Validation Accuracy	Testing Accuracy ± 95% C.I.	Train/Validation Accuracy	Testing Accuracy ± 95% C.I.
DRL_1_	17 waveforms + down sampled by 32	136 × 256	98.7%/85.8%	86.1 ± 1.95%	98.3%/82.3%	82.1 ± 2.16%
DRL_2_	4 waveforms + down sampled by 32	32 × 256	96.0%/85.3%	87.8 ± 1.85%	94.1%/81.2%	81.3 ± 2.20%
DRL_3_	4 waveforms + down sampled by 16	32 × 512	95.3%/84.5%	85.9 ± 1.96%	93.8%/82.1%	80.0 ± 2.26%

The testing accuracy for each model configuration is reported with associated 95% confidence intervals (C.I.) accounting for the limited testing dataset size. These confidence intervals were calculated by treating the outcomes of individual classification models as independent and identically distributed (I.I.D.) Bernoulli random variables with probability *p* of making the correct prediction (De Veaux et al., 2005). We observe from Table 1 that DRL models show consistent testing performance for all three input combinations as evidenced by overlapping confidence intervals, albeit with subtle changes in the training and validation accuracies. Specifically, the large difference between training and validation scores for all three model configurations show signs of overfitting. Additionally, we observe that the down sampling factor of 16 in DRL_3_ shows a slight reduction in the point estimate for testing accuracy, possibly due to the recurrent layers having to essentially process double the amount of time steps compared to other inputs. However, note that the results of each configuration are still well within the error margins of each other and therefore requires further analysis with a larger testing set to form conclusions. Overall, DRL classification models exhibit better performance in cavity classification compared to fault classification.

### Convolutional Neural Network

**FIGURE 8:**
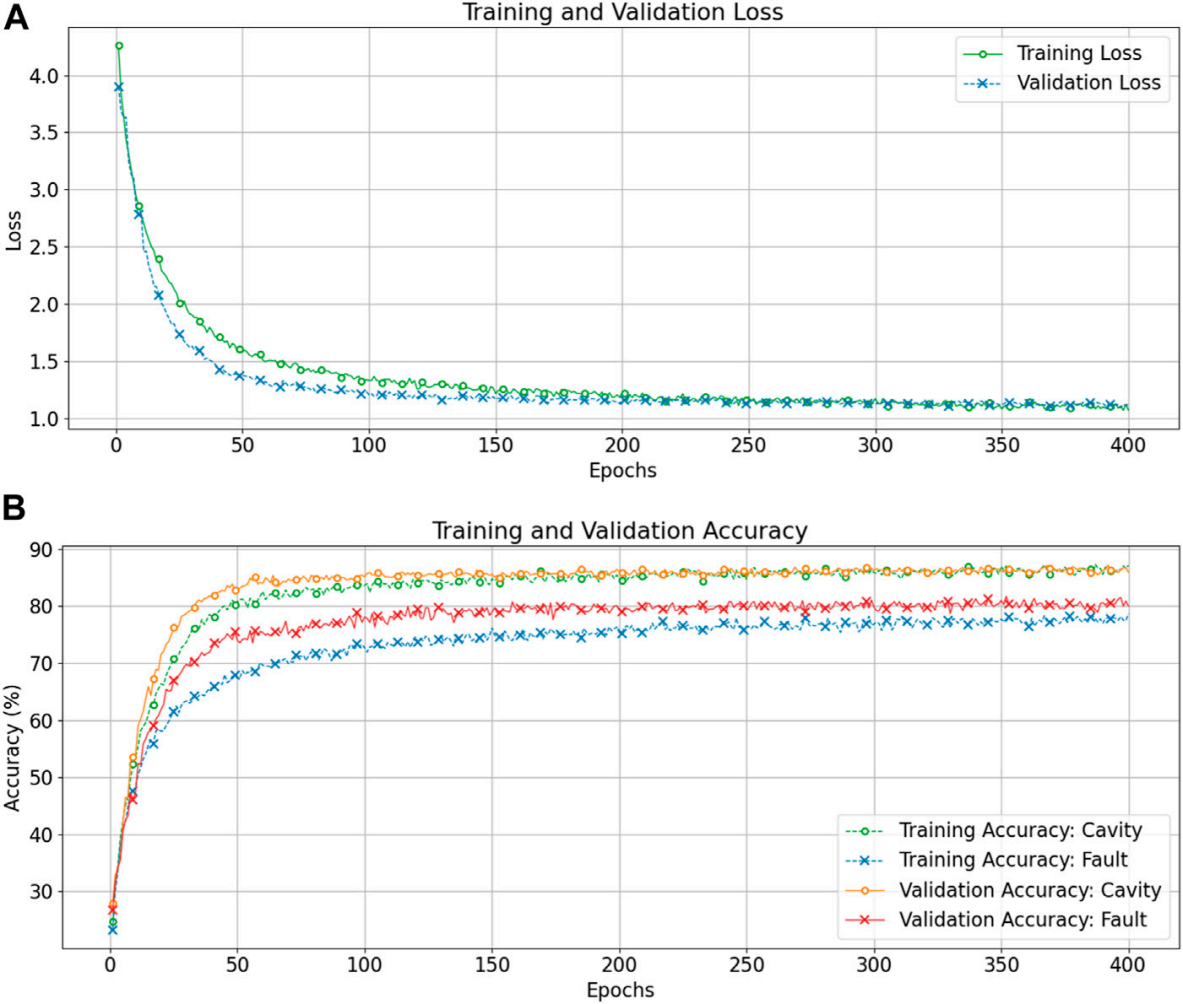
Training and validation loss curves **(A)**, and training and validation accuracy curves **(B)** as a function of the epochs for the CNN with raw signal direct conversion input method with input size ( 136 signals × 256 time steps).

We report and compare performance for different input sizes of the CNN and discuss the impact of input representation on the accuracy for cavity and fault recognition. CNN model analysis is conducted following the same data split method outlined in *Deep Recurrent Learning*. The training for CNN is performed for 400 epochs with batch-wise gradient updates. We obtain validation results at each epoch to verify proper training. Figure 8 shows training and validation loss/accuracy characteristics for the CNN with the direct conversion method (input size: 136 × 256).

The training and validation loss curves show consistent improvement over training epochs, though it requires a larger number of epochs compared to DRL models. The CNN model with DSC input exhibits slow but consistent convergence with no visible overfitting. The validation accuracy curves show a similar trend. Table 2 summarizes the results obtained through the CNN model with the DSC technique for input. Note that the computational efficiency of a CNN allows us to use larger input with all 136 signals easily (17 signals/cavity × 8 cavities). Therefore, we consider input combinations with down sampling factors 8, 16, and 32 to construct input images of size 136 × (1,024, 512, 256) denoted as CNN_1,_ CNN_2,_ and CNN_3_, respectively.

**TABLE 2:** Results of CNN model applying to raw data direct representation input. Performance after 400 epochs.

Model	Input combination	CNN input size	Cavity identification		Fault classification	
			Train/Validation Accuracy	Testing Accuracy ± 95% C.I.	Train/Validation Accuracy	Testing Accuracy ± 95% C.I.
CNN_1_	17 waveforms + down sampled by 8	136 × 1,024	92.6%/85.6%	85.8 ± 1.97%	89.1%/79.4%	78.6 ± 2.31%
CNN_2_	17 waveforms + down sampled by 16	136 × 512	88.6%/86.8%	87.2 ± 1.89%	82.9%/81.8%	78.4 ± 2.32%
CNN_3_	17 waveforms + down sampled by 32	136 × 256	87.1%/85.9%	86.8 ± 1.91%	78.7%/80.1%	76.5 ± 2.39%

While the CNN with DSC inputs exhibits consistent performance across different down sampling factors, the largest input size of 136 × 1,024 seems to cause the model to overfit slightly. As before, these confidence intervals are generated by considering each outcome of a given model architecture as an I.I.D. Bernoulli random variable with probability *p* of making the correct prediction. This slight overfitting is evident by the larger difference between training and validation data accuracy compared to other, smaller input configurations. However, all three input combinations show consistent testing accuracies with highly overlapping confidence intervals.

### Performance Comparison

We conduct a performance comparison between the two DL architectures in order to determine the feasibility for future deployment in CEBAF for online inference. The comparison is based on the model’s accuracy in identifying the C100 cavity that failed, identifying the type of RF fault, and the speed of inference for both tasks (i.e. the time taken for a raw example to be processed by a trained model). Statistically speaking, none of CNN models stands out as superior than the others. The same is true for the DRL models. Nevertheless, for the sake of comparison, we choose CNN_2_ and DRL_2_. Additionally, we compare the performance of these selected DL models with the current state-of-the-art ML models developed to perform the same tasks, in terms of classification accuracy as well as processing runtime.

We measure runtime performance across two operations, pre-processing (down sampling/feature extraction) and model inference (classification). As previously noted, the data standardization is common for all classification methods, and therefore excluded from runtime comparison. Pre-processing steps are performed on the CPU for all models. Inference occurs on the CPU for the ML model and GPU for the DL models. All calculations are performed on the same high-end mobile workstation (laptop), equipped with a hexa-core Intel Xeon E-2276M CPU and NVIDIA Quadro RTX 4000 GPU so that a reasonable baseline can be achieved for performance comparisons across CPU and GPU configurations of similar cost. More detailed timing studies are required to definitively pick the fastest model given a specific operating environment.

The ML pipeline implements a feature extraction scheme based on autoregressive analysis of each signal to obtain 192 features representing each event (Tennant et al., 2020). For the purpose of comparison, we implement this feature extraction scheme for each RF signal parallelized across a six-core CPU using six workers. The ML model uses two independent random forest classifiers (Breiman, 2001) (with CPU based parallelization using 6 workers) for cavity identification and fault classification tasks. (Appendix B provides a brief description of the ML pipeline). The signal down sampling routine for DL models is also optimized similarly for the given CPU. Table 3 summarizes the testing classification accuracies and runtime for each model.

**TABLE 3:** Performance comparison between best performing DRL, CNN, and ML models. The runtime for the ML pipeline is performed using CPU-based parallelization while the CNN and DRL models are performed on a GPU.

Model	Input size		Cavity identification	Fault classification	Runtime CPU: Intel XEON hexa-core CPU @ 2.8 GHz (laptop) GPU: NVIDIA Quadro RTX 4000 (laptop) (For 1 example in seconds)		
	Raw	Model	Testing Accuracy ± 95% C.I.	Testing Accuracy ± 95% C.I.	Down sampling/feature extraction	Classification	Total
ML pipeline	32 × 8,192	1 × 192	88.0 ± 1.83%	86.7 ± 1.92%	NA/0.063	0.078	0.141
DRL_2_	32 × 8,192	32 × 256	87.8 ± 1.85%	81.3 ± 2.20%	0.008/NA	0.031	0.039
CNN_2_	136 × 8,192	136 × 512	87.2 ± 1.89%	78.4 ± 2.32%	0.020/NA	0.016	0.036

**FIGURE 9:**
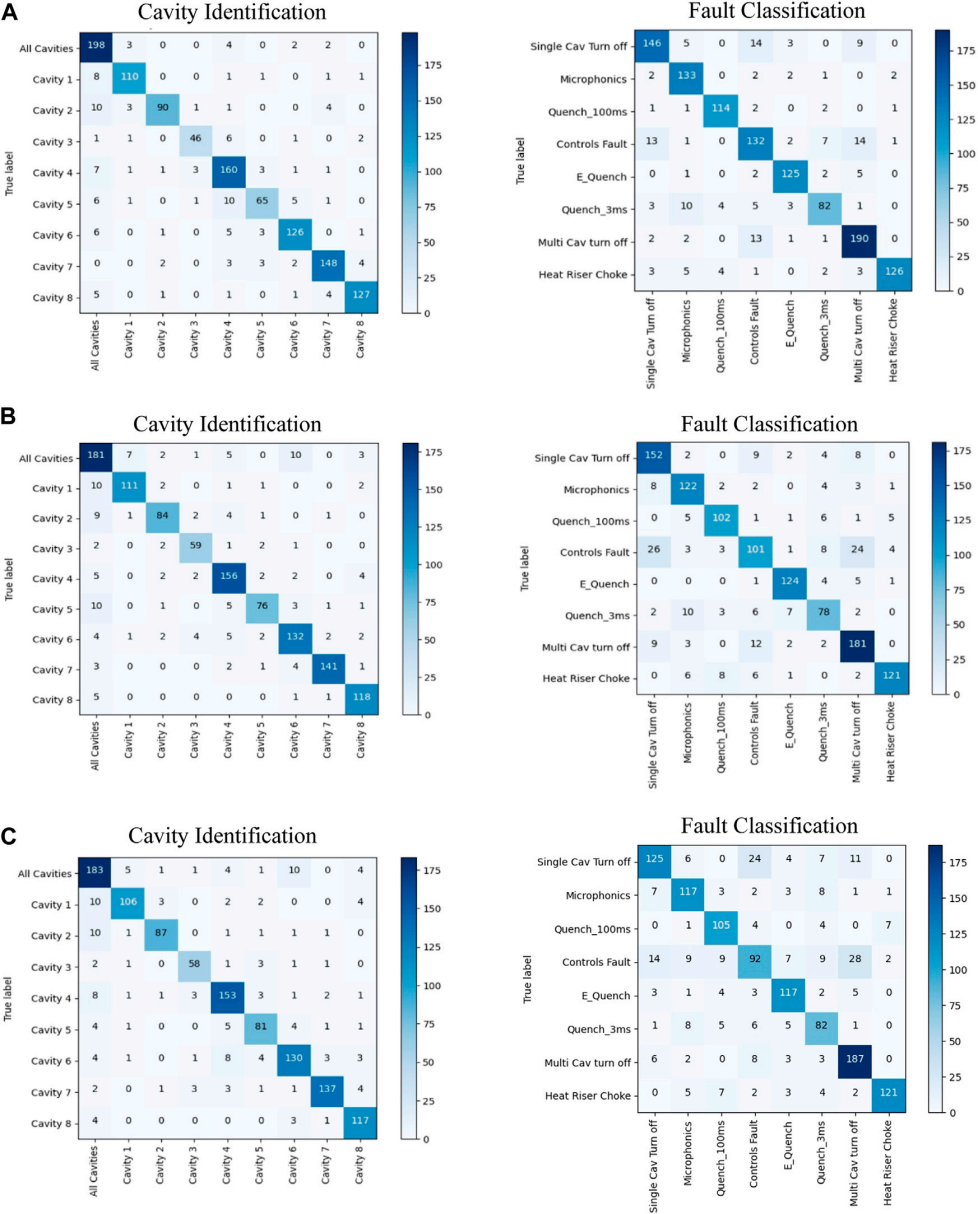
The cavity identification and fault classification confusion matrices for the **(A)** ML pipeline, **(B)** DRL model, and **(C)** CNN with direct conversion input model for testing data.

The classification accuracy comparison in Table 3 shows that while all three models perform cavity classification with similar accuracy, the ML pipeline achieves higher fault classification accuracy than the DL models. A more in-depth look into the classification outputs of each model can be obtained by inspecting the corresponding confusion matrices for cavity identification and fault classification as shown in Figure 9. The fault classification confusion matrices of DL models in Figures 9A,B show that significant misclassifications have occurred between “Controls Fault”, “Single Cavity Turn Off”, and “Multi-Cavity Turn Off” fault types. This indicates that further analysis into the characteristics of these fault types is required to understand the causes that inhibits DL performance. ML models perform comparatively well in the presence of limited data and noise, owing to the human engineered feature extraction and a choice of classification algorithms that are more robust against overfitting. Even though the fault identification confusion matrix of the ML model (Figure 9A) show that a certain confusion between “Controls Fault”, “Single Cavity Turn Off”, and “Multi-Cavity Turn Off” still exist, it is significantly less pronounced compared to DL models. Supplementary Appendix Tables SAC1, C2 in Appendix C further summarizes the DL and ML models fault class specific performance in terms of precision, recall rate, and F1-score.

The DL models show an advantage over the ML pipeline in terms of runtime. While DL models process raw RF signals as input and produce cavity and fault classifications simultaneously, the ML pipeline is slowed by the autoregressive (AR) feature extraction step with a computational cost of 0.063 s per example. And in fact, the computation cost of the classification with the ML model is 0.047 s slower compared to the slowest inference with DL. These results are suggestive that the DL models would result in significantly faster runtimes, however the final operating environment would heavily influence relative runtimes.

## Summary and Conclusion

This work investigates the ability of DL models to automate the process of cavity and fault classification using RF signals as an alternative to a conventional ML pipeline. We have developed custom DL models based on recurrent neural network and convolutional neural network architectures. The proposed DRL model draws from the ability to process RF time-series directly through its bi-directional LSTM layers to identify the faulty cavity and to classify the fault type simultaneously. The CNN-based architecture is typically used in computer vision applications. Consequently, this work utilizes a direct conversion method to provide a straightforward representation by arranging the raw RF signals from an example in a 2D array. This results in a lossless conversion yielding an input with a width and height corresponding to the length, and the number of RF signals, respectively.

Models that were developed on the DRL and CNN architectures and explored different data input sizes exhibit similar testing accuracies across both the cavity identification and fault classification tasks. In comparison to the ML pipeline—which is currently deployed and in use at CEBAF—the fault classification accuracy of the DL models suffer. We posit that this is due to the limitations in the number of examples, and representation of certain fault types in the current dataset, along with possible labeling noise. We continue to collect more data and will revisit this hypothesis in future studies. Nevertheless, the DL models achieve a sufficiently high accuracy so as to make them a beneficial tool, with the added benefit of having faster runtimes compared to ML. They, therefore, are a viable alternative to the conventional ML system for future autonomous tasks where runtime represents a key performance metric.

## Data Availability

The data analyzed in this study is subject to the following licenses/restrictions: Raw data for the study were generated at Jefferson Lab. Derived data supporting the findings of this study maybe provided upon request. Requests to access these datasets should be directed to Chris Tennant: tennant@jlab.org.

## References

[B1] Beveridge S. Nelson C. R. (1981). A New Approach to Decomposition of Economic Time Series into Permanent and Transitory Components with Particular Attention to Measurement of the 'business Cycle'. J. Monetary Econ. 7 (2), 151–174. 10.1016/0304-3932(81)90040-4

[B2] Breiman L. (2001). Random Forests. Machine Learn. 45, 5–32. 10.1023/a:1010933404324

[B3] Caiado J. Crato N. Peña D. (2006). A Periodogram-Based Metric for Time Series Classification. Comput. Stat. Data Anal. 50 (10), 2668–2684. 10.1016/j.csda.2005.04.012

[B4] Cho J. Hwang H. (2020). Spatio-temporal Representation of an Electoencephalogram for Emotion Recognition Using a Three-Dimensional Convolutional Neural Network. Sensors 20, 3491. 10.3390/s20123491 PMC7349167 32575708

[B5] Chua K. C. Chandran V. Acharya U. R. Lim C. M. (2010). Application of Higher Order Statistics/spectra in Biomedical Signals-A Review. Med. Eng. Phys. 32 (7), 679–689. 10.1016/j.medengphy.2010.04.009 20466580

[B6] De Veaux R. D. Velleman P. F. Bock D. E. Vukov A. M. Wong A. C. (2005). Stats: Data and Models. Boston, MA: Pearson/Addison Wesley Boston.

[B7] Eren L. Ince T. Kiranyaz S. (2019). A Generic Intelligent Bearing Fault Diagnosis System Using Compact Adaptive 1D CNN Classifier. J. Sign Process. Syst. 91, 179–189. 10.1007/s11265-018-1378-3

[B8] Géron A. (2019). Hands-on Machine Learning with Scikit-Learn, Keras, and TensorFlow: Concepts, Tools, and Techniques to Build Intelligent Systems. Sebastopol, CA: O'Reilly Media.

[B9] Kingma D. P. Ba J. (2014). Adam: A Method for Stochastic Optimization. Ithaca, NY: arXiv preprint arXiv:1412.6980.

[B10] Krizhevsky A. Sutskever I. Hinton G. E. (2012). “Imagenet Classification with Deep Convolutional Neural Networks,” in Advances in Neural Information Processing Systems, 1097–1105.

[B11] LeCun Y. Bottou L. Bengio Y. Haffner P. (1998). Gradient-based Learning Applied to Document Recognition. Proc. IEEE 86 (11), 2278–2324. 10.1109/5.726791

[B12] Malhotra P. Vig L. Shroff G. Agarwal P. (2015). “Long Short Term Memory Networks for Anomaly Detection in Time Series,” in ESANN 2015 proceedings, European Symposium on Artificial Neural Networks, Computational Intelligence and Machine Learning. Bruges, Belgium. April 22–24, 2015. i6doc.com publ. (Presses universitaires de Louvain), 89.

[B13] Meidinger E. E. (1980). Applied Time Series Analysis for the Social Sciences. New York City, NY: Sage Publications.

[B14] Paszke A. (2019). “Pytorch: An Imperative Style, High-Performance Deep Learning Library,” in Advances in Neural Information Processing Systems, 8026–8037.

[B15] Reece C. E. (2016). Continuous Wave Superconducting Radio Frequency Electron Linac for Nuclear Physics Research. Phys. Rev. Accel. Beams 19 (12), 124801. 10.1103/PhysRevAccelBeams.19.124801

[B16] Schuster M. Paliwal K. K. (1997). Bidirectional Recurrent Neural Networks. IEEE Trans. Signal. Process. 45 (11), 2673–2681. 10.1109/78.650093

[B17] Shoeb A. H. Guttag J. V. (2010). “Application of Machine Learning to Epileptic Seizure Detection,” in Proceedings of the 27th International Conference on Machine Learning (Haifa, Israel: ICML-10), 975–982.

[B18] Shumway R. H. Stoffer D. S. Stoffer D. S. (2000). Time Series Analysis and its Applications. Springer.

[B19] Simonyan K. Zisserman A. (2014). in Very Deep Convolutional Networks for Large-Scale Image Recognition (Ithaca, NY: arXiv preprint arXiv:1409.1556).

[B20] Tennant C. Carpenter A. Powers T. Shabalina Solopova A. Vidyaratne L. Iftekharuddin K. (2020). Superconducting Radio-Frequency Cavity Fault Classification Using Machine Learning at Jefferson Laboratory. Phys. Rev. Accel. Beams 23, 114601. 10.1103/physrevaccelbeams.23.114601 PMC8762208 35047766

[B21] Ullah I. Hussain M. Qazi E.-u. -H. Aboalsamh H. (2018). An Automated System for Epilepsy Detection Using EEG Brain Signals Based on Deep Learning Approach. Expert Syst. Appl. 107, 61–71. 10.1016/j.eswa.2018.04.021

[B22] Vidyaratne† L. Carpenter A. Suleiman R. Tennant C. Turner D. Iftekharuddin K. Monibor Rahman Md. “Initial Studies of Cavity Fault Prediction at Jefferson Laboratory,” in Presented at the 18th International Conference on Accelerator and Large Experimental Physics Control Systems (ICALEPCS 2021) (Shanghai, China. October 14-22, 2021, Paper presented.

[B23] Wei X. Zhou L. Chen Z. Zhang L. Zhou Y. (2018). Automatic Seizure Detection Using Three-Dimensional CNN Based on Multi-Channel EEG. BMC Med. Inform. Decis. Mak 18, 111–180. 10.1186/s12911-018-0693-8 30526571 PMC6284363

[B24] Zhao X. Zhang H. Zhu G. You F. Kuang S. Sun L. (2019). A Multi-Branch 3D Convolutional Neural Network for EEG-Based Motor Imagery Classification. IEEE Trans. Neural Syst. Rehabil. Eng. 27, 2164–2177. 10.1109/TNSRE.2019.2938295 31478864

