# Supplementary material for: Deep Learning Based Superconducting Radio-Frequency Cavity Fault Classification at Jefferson Laboratory
Source: Front Artif Intell. 2022 Jan 3;4:718950. doi: 10.3389/frai.2021.718950 (PMC8762208; doi:10.3389/frai.2021.718950)
Supplement: Supplementary file 1 [file DataSheet1.docx]

Supplementary Material

**Supplementary Appendix A: RF Signals and Description**

Table SA1 provides a description of the 17 RF signals from each C100 cavity recorded by our data acquisition system.

Table SA1: List of the 17 diagnostic RF signals recorded by the DAQ system along with a brief description of each

| **Signal Name** | **Description** |
| --- | --- |
| IMES | probe in phase voltage magnitude (analog-to-digital converter (ADC) counts) |
| QMES | probe quadrature voltage magnitude (ADC counts) |
| GMES | measured gradient (MV/m) |
| PMES | measured phase (degrees) |
| IASK | drive signal in phase voltage magnitude (ADC counts) |
| QASK | drive voltage quadrature voltage (ADC counts) |
| GASK | sqrt(IASK^2^ + QASK^2^) × (10 / ADC maximum) |
| PASK | $\tan^{-1} (IASK/QASK)$ |
| CRFP | forward power (kW) |
| CRFPP | forward power measured phase (degrees) |
| CRRP | reflected power (kW) |
| CRRPP | reflected power phase (degrees) |
| GLDE | gradient error (MV/m) |
| PLDE | phase error (degrees) |
| DETA2 | detune angle (CRFPP − PMES − TDOFF) |
| CFQE2 | cavity frequency error (degrees) |
| DFQES | discriminator frequency error |

**Supplementary Appendix B: Summary of ML Pipeline Used for Comparison**

The overall ML pipeline used for performance comparison is summarized in Fig. B1. The detailed schematics with training and testing methods associated with the ML pipeline is presented in [1]. Note that the ML pipeline outlined by Tennant et al. [1] is recreated in this study for a fair comparison with DL models using the same data. In summary, an input example for the ML pipeline constitutes of 32 waveforms (GMES, GASK, CRFP, DETA2 collected from each of the 8 cavities in the cryomodule). Each waveform undergoes data normalization as described in Eq. (1). Feature extraction is performed using an approach based on autoregressive modeling. Finally, the extracted features are used by two trained random forest classification models to determine the corresponding cavity and fault classes of the example.

**
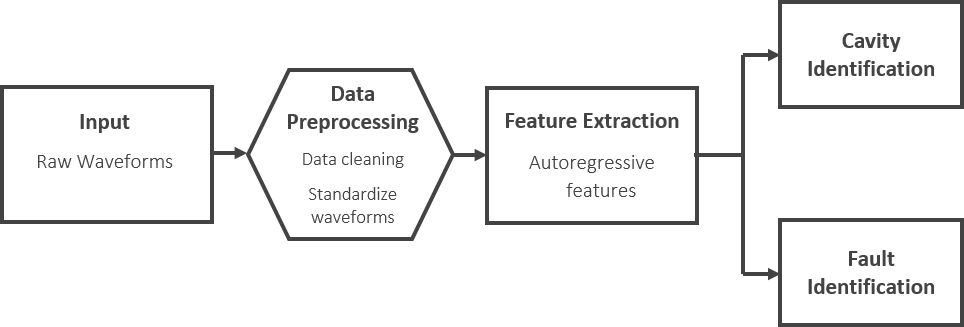
**

Fig SAB1: The ML pipeline introduced by Tennant et al. [1] for cavity identification and fault classification.

**Supplimentery Appendix C: Class specific performance measures for best performing models**

Tables SAC1 and SAC2 summarizes the class specific performance characteristics of the best performing DRL, CNN, and ML models for testing data. The performance figures correspond to the accuracies provided in Table 3 and Fig 9.

Table SAC1: Fault class specific performance measures for the best performing DL and ML models.

|  | **DRL Model** | | | **CNN Model** | | | **ML Model** | | |
| --- | --- | --- | --- | --- | --- | --- | --- | --- | --- |
|  | Precision (%) | Recall (%) | F1 Score (%) | Precision (%) | Recall (%) | F1 Score (%) | Precision (%) | Recall (%) | F1 Score (%) |
| Single Cav Turn off | 77.2 | 85.9 | 81.3 | 80.1 | 70.6 | 75.1 | 85.9 | 82.5 | 84.1 |
| Microphonics | 80.8 | 85.9 | 83.3 | 78.5 | 82.4 | 80.4 | 84.2 | 93.7 | 88.7 |
| Quench_100ms | 86.4 | 84.3 | 85.4 | 78.9 | 86.8 | 82.7 | 93.4 | 94.2 | 93.8 |
| Controls Fault | 73.2 | 59.4 | 65.6 | 65.2 | 54.1 | 59.2 | 77.2 | 77.6 | 77.4 |
| E_Quench | 89.9 | 91.9 | 90.8 | 82.4 | 86.7 | 84.5 | 91.9 | 92.6 | 92.3 |
| Quench_3ms | 73.6 | 72.2 | 72.9 | 68.9 | 75.9 | 72.2 | 84.5 | 75.9 | 80.0 |
| Multi Cav turn off | 80.1 | 86.6 | 83.2 | 79.6 | 89.5 | 84.2 | 85.6 | 90.9 | 88.2 |
| Heat Riser Choke | 91.7 | 84.0 | 87.7 | 92.4 | 84.0 | 88.0 | 96.9 | 87.5 | 92.0 |

Table SAC2: Cavity class specific performance measures for the best performing DL and ML models.

|  | **DRL Model** | | | **CNN Model** | | | **ML Model** | | |
| --- | --- | --- | --- | --- | --- | --- | --- | --- | --- |
|  | Precision (%) | Recall (%) | F1 Score (%) | Precision (%) | Recall (%) | F1 Score (%) | Precision (%) | Recall (%) | F1 Score (%) |
| All Cavities | 79.0 | 86.6 | 82.6 | 80.6 | 87.6 | 83.9 | 82.2 | 94.7 | 88.0 |
| Cavity 1 | 92.5 | 87.4 | 89.9 | 91.4 | 83.5 | 87.2 | 92.4 | 90.2 | 91.3 |
| Cavity 2 | 88.4 | 82.4 | 85.3 | 93.5 | 85.3 | 89.2 | 94.7 | 82.6 | 88.2 |
| Cavity 3 | 86.8 | 88.1 | 87.4 | 87.9 | 86.6 | 87.2 | 90.2 | 80.7 | 85.2 |
| Cavity 4 | 87.2 | 90.2 | 88.6 | 86.4 | 88.4 | 87.4 | 83.8 | 90.4 | 87.0 |
| Cavity 5 | 89.4 | 78.4 | 83.5 | 84.4 | 83.5 | 83.9 | 86.7 | 73.0 | 79.3 |
| Cavity 6 | 86.3 | 85.7 | 86.0 | 86.1 | 84.4 | 85.2 | 91.3 | 88.7 | 90.0 |
| Cavity 7 | 96.6 | 92.8 | 94.6 | 93.8 | 90.1 | 91.9 | 91.9 | 91.4 | 91.6 |
| Cavity 8 | 90.1 | 94.4 | 92.2 | 87.3 | 93.6 | 90.3 | 94.1 | 91.4 | 92.7 |
